# A Stability-Indicating HPLC Method for the Determination of Fingolimod in Pharmaceutical Dosage Forms

**DOI:** 10.3797/scipharm.1408-08

**Published:** 2014-09-23

**Authors:** Effat Souri, Mohammad Zargarpoor, Siavash Mottaghi, Reza Ahmadkhaniha, Abbas Kebriaeezadeh

**Affiliations:** 1 Department of Medicinal Chemistry, Faculty of Pharmacy and Drug Design and Development Research Center, Tehran University of Medical Sciences, Tehran 141556451, Iran; 2 Department of Human Ecology, School of Public Health, Tehran University of Medical Sciences, Tehran 1417613151, Iran; 3 Department of Pharmacoeconomy and Pharmaceutical Administration, Faculty of Pharmacy, Tehran University of Medical Sciences, Tehran, Iran

**Keywords:** Fingolimod, HPLC, Stability-Indicating, Stress degradation

## Abstract

Fingolimod is an immunosuppressive agent which is used for the prophylaxis of organ transplantation rejection or multiple sclerosis treatment. In this study, systematic forced degradation studies on fingolimod bulk powder were performed to develop a stability-indicating HPLC method. Separation of fingolimod and its degradation products was achieved on a Nova-Pak C_8_ column. The mobile phase was a mixture of potassium dihydrogenphosphate 50 mM (pH 3.0) and acetonitrile (45:55, v/v) at a flow rate of 1 ml/min. The proposed method was linear in the range of 0.125–20 μg mL^−1^. The within-day and between-day coefficients of variation were in the range of 0.6–1.2%. The developed method was successfully applied for the determination of the fingolimod amount in pharmaceutical dosage forms.

## Introduction

**Fig. 1:**
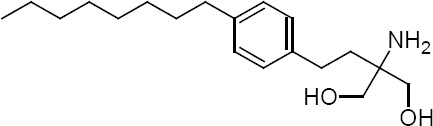
Chemical structure of fingolimod

Fingolimod, 2-amino-2-[2-(4-octylphenyl)ethyl]propane-1,3-diol (Figure 1), is a synthetic compound with immunosuppressive effects which is used for the treatment of multiple sclerosis and also rejection prophylaxis after organ transplantation [1, 2]. Fingolimod is metabolized to its phosphorylated form which could reduce peripheral lymphocytes through its interaction with sphingosine-1-phosphate (SIP) receptors on lymphocytes [3]. Activation of SIP receptors is necessary for the circulation of leukocytes from lymph nodes to other organs [4].

Few LC-MS methods have been reported before for the determination of fingolimod in biological fluids [5–8]. According to the literature survey, there are two published HPLC methods for the determination of fingolimod in pharmaceutical dosage forms [9, 10]. In the first method [9], a gradient HPLC mode with a C18 column at 40°C was used for chromatographic separation and a retention time of about 9 min was observed. The linearity range was reported to be 20–150 μg mL^−1^. The other method [10] was also based on an ion-pair gradient mode with a column temperature of 45°C which resulted in a retention time of 10 min. Fingolimod is not officially available in any pharmacopeia and there is still a need for accurate and simple HPLC methods for the determination of fingolimod in pharmaceutical dosage forms.

Our study focused on the development and validation of a simple and fast stability-indicating HPLC method which could be used for the determination of fingolimod in the presence of its degradation products. These degradation products resulted from the storage of the drug under stress degradation conditions recommended by the International Conference on Harmonization (ICH). Although structural characterization of the degradation products was not performed, this study could be a valuable, validated analytical method for the determination of fingolimod in the presence of its degradation products.

## Results and Discussion

### Chromatographic Conditions

**Fig. 2:**
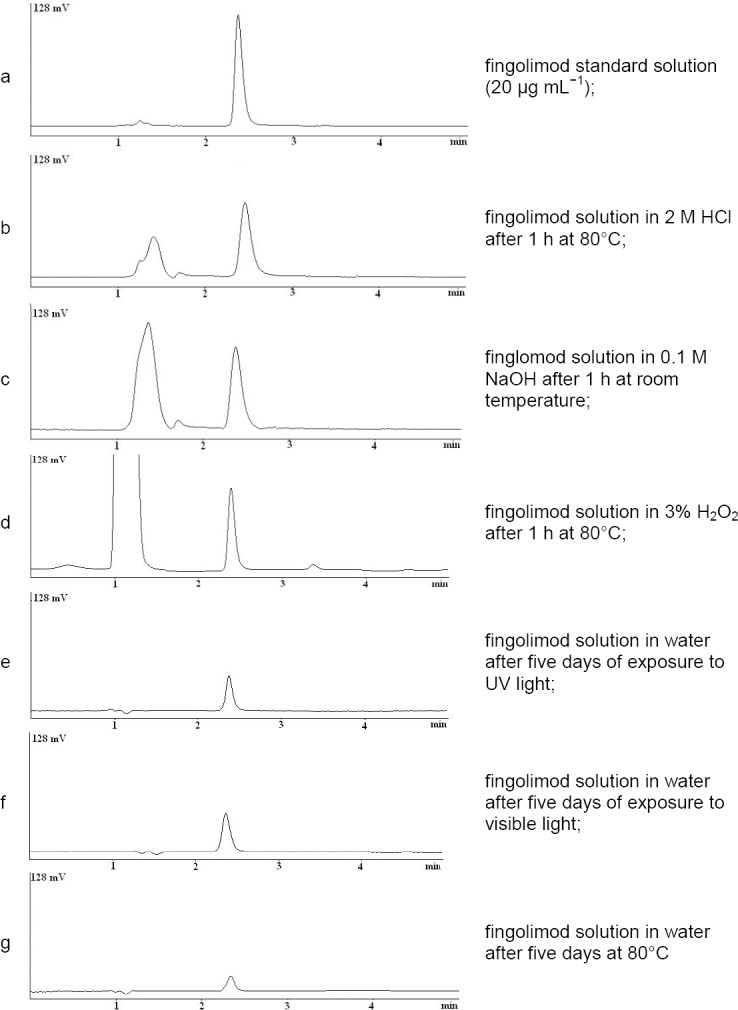
Typical chromatograms obtained from stability studies of fingolimod.

Using a Nova-Pak C_18_ column and varied proportions of the organic phase and buffer solution, a symmetrical peak with acceptable retention time was not achieved. Better peak shape and retention times were observed by using a Nova-Pak C_8_ column for separation. The representative chromatograms obtained by using the C_8_ column and a mixture of KH_2_PO_4_ 50 mM (pH 3.0) and acetonitrile (45:55) as the mobile phase are illustrated in Figure 2. The retention time of fingolimod was about 2.4 min, which is very short. In other reported HPLC methods [9, 10] using the gradient mode, the reported retention time of fingolimod was about 10 min.

To assess the system suitability of the HPLC method, six replicate analyses were performed at a concentration value of 10 µg mL^−1^. The chromatographic parameters were calculated and compared with the accepted criteria (Table 1).

**Tab. 1:**
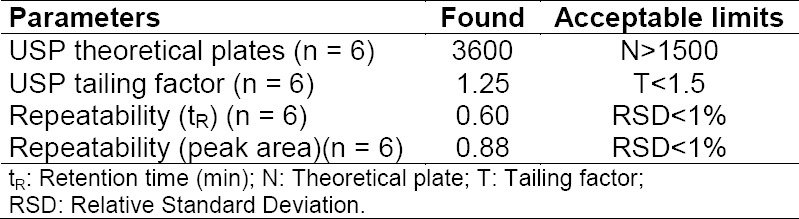
System suitability parameters

### Linearity

To evaluate the linearity of the proposed method, calibration curves were constructed by plotting the resulting peak area of the calibration solutions in the range of 0.125–20 µg mL^−1^ versus the fingolimod concentration. The statistical data of six series of calibration curves are indicated in Table 2. The linearity of the method within the range of 0.125–20 µg mL^−1^ was satisfactory for quality control purposes. The standard deviation of the slope and intercept was acceptable for replicate injections. Also, the LOQ of the HPLC method was appropriate for the determination of fingolimod in the assay solutions or dissolution mediums.

**Tab. 2:**
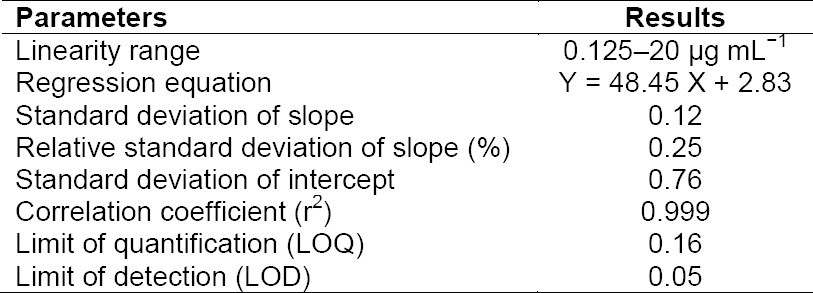
Statistical data of the calibration curves of fingolimod (n=6)

### Accuracy and Precision

The data obtained for within-day and between-day accuracy and precision given in Table 3 confirm sufficient repeatability of the proposed method. The CV values were within 0.6–1.2% and the error values were between 0.01–1.2%, which is acceptable for the determination of active ingredients in pharmaceutical dosage forms according to the guidelines.

**Tab. 3:**
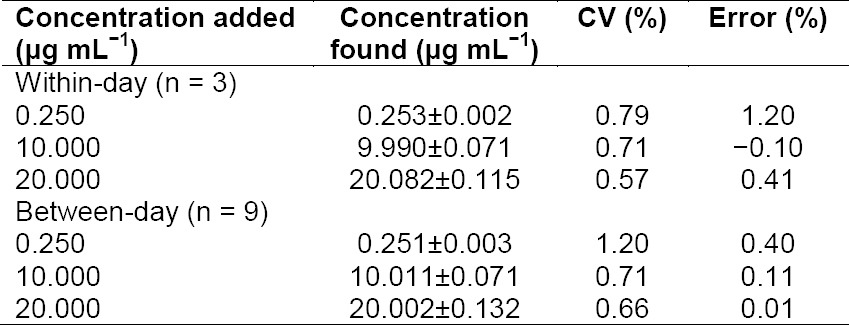
Precision and accuracy of the method for the determination of fingolimod (three sets for three days)

### Robustness

Under the varied chromatographic conditions (organic phase composition and pH value of the buffer solution), the fingolimod peak was separated from the degradation peaks and there was no significant change in the peak area, which illustrated the robustness of the proposed method.

### Stability

No significant changes in the fingolimod content were observed in the stock standard solutions after seven days in the refrigerator (recovery 98.7±0.2%) or working standard solutions after 24 h at room temperature (recovery 99.7±0.7%).

### Application of the Proposed Method

The developed HPLC method was applied for the determination of fingolimod in its pharmaceutical dosage forms. The results of triplicate analysis showed that the amount of fingolimod was 0.49±0.03 mg/capsule which was in great accordance with the label claim (0.5 mg).

### Relative Recovery

Standard addition was conducted on a capsule sample to find out the relative recovery of fingolimod. The recovery from the dosage forms was found to be 100.12±0.94, which showed insignificant interference of the excipients by the proposed HPLC method.

### Degradation Studies

The degradation results are summarized in Table 4. Upon treatment of fingolimod under different stress conditions, it was shown that fingolimod was labile in all of the stress degradation tests studied.

**Tab. 4:**
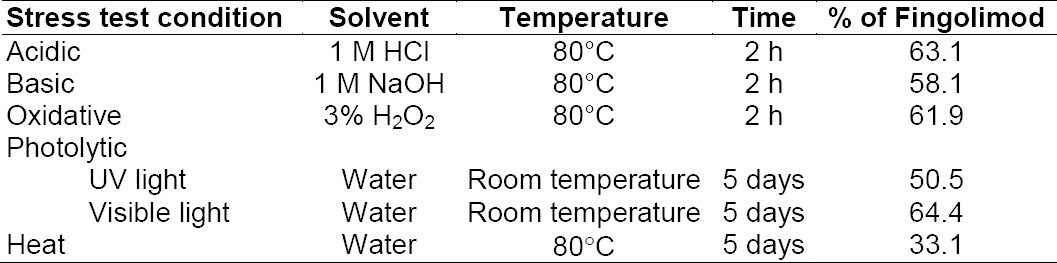
The results of the stress degradation tests on fingolimod bulk powder using different conditions

#### Acid Degradation

Figure 2a shows the decomposition of fingolimod under acidic stress conditions. About 37% degradation was observed after 2 h exposure to 1 M HCl at 80°C. At a lower strength of HCl or a lower temperature, the observed degradation was very low. A broad degradation peak was observed in acidic conditions with the retention time of about 1.2 min.

#### Base Degradation

Significant degradation was also observed (about 42%) under alkaline conditions using 1 M NaOH at 80°C for 2 h with a main degradation product at the retention time of about 1.2 min (Figure 2c). Also, when using milder conditions, no significant degradation was observed.

#### Oxidative Degradation

Upon treatment of fingolimod with 3% hydrogen peroxide, about 38% degradation was achieved. A small peak for the degradation product at the retention time of 3.4 min was found (Figure 2d).

#### Photolytic Degradation

Significant degradation of the fingolimod solution under photolytic conditions showed the photosensitivity of the drug. The fingolimod concentration fell to about 50% or 64% after five days of exposure to UV light or visible light, respectively. No major peak was observed in these conditions (Figures 2e and 2f). Fingolimod bulk powder was shown to be relatively stable under photolytic conditions [9, 10].

#### Thermal Degradation

Fingolimod solution was also very labile upon exposure to heat. About 67% degradation was observed after five days of exposure to heat (Figure 2g). Fingolimod bulk powder was shown to be relatively stable under thermal conditions [9, 10], but the fingolimod solution was very labile.

## Experimental

### Chemicals

Fingolimod bulk powder and Fingolimod 0.5 mg capsules were kindly provided by Osvah Pharmaceutical Compary, Tehran, Iran. HPLC grade solvents and analytical grade compounds were purchased from Merck (Darmstadt, Germany).

### Instrumentation

The HPLC system consisted of a pump (Model 515), a UV-Vis detector (Model 486), and an autosampler (Model 717) from Waters (Milford, USA). The HPLC data were processed using multi-channel Chrom&Spec software, version 1.5 x. Thermal degradation was performed in a Melag dry air oven (Germany). A 100 W tungsten (visible light) and a low pressure mercury lamp (UV light) were used for the photolytic degradation studies.

### Chromatographic Conditions

Chromatographic separation was carried out on a Nova-Pak C_8_ column by using a mixture of KH_2_PO_4_ 50 mM (pH 3.0) and acetonitrile (45:55) at a flow rate of 1 ml min^−1^ as the mobile phase. The wavelength for the UV detection was 220 nm. The mobile phase was filtered through a 0.45 µm Teflon membrane filter (Millipore, Milford, USA) and sonicated for 10 min for degassing prior to use.

### Standard Solutions

The stock standard solution of fingolimod (500 µg mL^−1^) was prepared in methanol. Working standard solutions were prepared after the dilution with the mobile phase. Six series of fingolimod calibration solutions at the concentration values of 0.125, 0.25, 0.5, 1, 2, 5, 10, 15, and 20 µg mL^−1^ were prepared from the stock standard solution by appropriate dilution with the mobile phase.

### Stability

The short-term stability of the drug solution was studied by keeping a working standard solution of fingolimod at room temperature for 24 h. Long-term stability was studied by keeping a stock standard solution in the refrigerator for seven days.

### Robustness

To find out the robustness of the chromatographic method, the effect of minor variations in the pH value (±0.2) and organic phase composition (±2%) of the mobile phase on chromatographic parameters were studied.

### Accuracy and Precision

Three quality control samples within the calibration range (0.25, 10, and 20 µg mL^−1^) were prepared in triplicate and injected into the HPLC system. The concentration of each sample was calculated using the constructed calibration curve. The within-day coefficient of variation and error were calculated. The same procedure was performed for three consecutive days to study the between-day precision and accuracy.

### Degradation Studies

In this study, acidic, basic, oxidative, thermal, and photolytic degradation of fingolimod were studied. Hydrolytic degradation studies were performed using a 100 µg mL^−1^ solution of fingolimod in 1 M HCl or 1 M NaOH solution and heating at 80°C. For oxidative degradation, the same concentration of fingolimod was used in 3% hydrogen peroxide and heating at 80°C for 2 h. One ml of the degraded solutions were diluted five times after neutralization (for acidic and alkaline solutions) with the mobile phase and injected into the HPLC system.

Thermal and photolytic degradation studies on fingolimod were conducted on a solution prepared from fingolimod bulk powder (100 µg mL^−1^) in a mixture of water and methanol (8:2, v/v) and exposed to heat or light.

Reference solutions at the same concentration levels were used to calculate the concentration of the remaining drug in each degradation condition.

### Application of the Method

The contents of ten fingolimod capsules were weighed and thoroughly mixed. An accurate amount of the powder equivalent to one capsule was transferred into a 10-mL volumetric flask and sonicated for 20 min with 8 mL of methanol. The flask was made up to volume with methanol. After filtration, the solution was diluted with mobile phase to obtain a concentration of 10 µg mL^−1^, and 20 μL of the resulting solution was injected into the HPLC system. The drug amount was calculated by comparing the peak area with a standard solution at the same concentration value.

## Conclusion

A rapid, simple, accurate, precise, and robust stability-indicating HPLC method has been developed for the determination of fingolimod in pharmaceutical dosage forms. The retention time of fingolimod was very low in the proposed HPLC method which enabled the determination of a lot of samples in a limited time without any interference from the excipients or degradation products. As there is no pharmacopeial method for the determination of fingolimod in dosage forms, the proposed method could be a useful method for quality control laboratories.
